# Characterization of viral determinants of herpes simplex virus type 1 pathogenesis by bioimagery

**Published:** 2011-01-10

**Authors:** P-A Rochette, A Pearson

**Affiliations:** 1 INRS-Institut Armand Frappier, Laval, Québec, Canada, H7V 1B7

## 

The human pathogen herpes simplex virus 1 (HSV-1) infects mucous membranes leading to cold sores, following which a latent infection is established in neurons of the trigeminal ganglia (TG). While the infection in healthy individuals is usually benign, viral encephalitis may sometimes occur. Furthermore, the infection can lead to severe illness in immunocompromised individuals, and can result in permanent neurological sequelae in newborns.

The UL24 protein of HSV-1 is highly conserved throughout the *Herpesviridae* family, and has been identified as a viral determinant of pathogenesis. In a murine model of ocular infection, a UL24-deficient virus exhibits a modest reduction in viral titers in the eye and a severe reduction of viral titers in the TG. A virus that does not express UL24 also exhibits defects in the establishment of latency and in the efficiency of viral reactivation from latency, as compared to a wild-type virus. Although UL24 is important for the pathogenesis of HSV-1, its exact role *in vivo* is unknown.

We hypothesized that UL24 is important for viral dissemination to the trigeminal ganglia following ocular infection. To investigate this possibility, we generated a recombinant strain of HSV-1 expressing a second generation red fluorescent protein (RFP), mCherry. The RFP expression cassette, driven by the eukaryotic CMV promoter, was inserted within the intergenic region between the viral genes *Us7* and *Us8*. This virus, v*Us7-8*mCherry, behaved similarly to the wild type virus (KOS) in cell culture as well as *in vivo*. We did not detect a loss of the RFP cassette over multiple rounds of viral replication in cell culture or following passage of the virus in mice. Following ocular infection, histological cross sections of eyes and TGs harvested three days post-infection were observed by confocal microscopy. Detection of mCherry enabled us to easily visualize infected cells in both the eye and in TG.

This virus will be a powerful tool to study the role of UL24 in viral dissemination. In the long term, results from this project will help us further our understanding of the molecular mechanisms involved in viral pathogenesis, and possibly lead to the development of new therapeutic strategies.

